# Supplementary material for: AlphaFold predicts the most complex protein knot and composite protein knots
Source: Protein Sci. 2022 Jul 13;31(8):e4380. doi: 10.1002/pro.4380 (PMC9278004; doi:10.1002/pro.4380)
Supplement: Supplementary file 2 — Appendix S1 We discuss several knot‐related statistics of this list and compare them to the PDB. Moreover, we discuss further topologically interesting proteins, which match the criteria introduced above but for which visual inspection implies potential unreliability. Furthermore, we discuss the ERRAT accuracy test for the composite knot Q4D5S2 for which the program flags a topologically relevant beta strand segment as likely to be structurally incorrect, although the latter is assigned a high degree of confidence by AlphaFold. Finally, the supplementary material contains a depiction of the per‐residue confidence scores by AlphaFold for the proteins in Figures 1–4 as well as a discussion on the structure alignment and sequence identity of the 31#31‐methyltransferases [file PRO-31-e4380-s002.pdf]

Supplementary Information:

AlphaFold predicts the most complex protein knot and composite protein knots

Maarten A. Brems[1,†], Robert Runkel[1,†], Todd O. Yeates[2,3], Peter Virnau[1,*]

[1] Department of Physics, Johannes Gutenberg University Mainz, Staudingerweg 9, 55128 Mainz, Germany.

[2] UCLA-DOE Institute for Genomics and Proteomics, University of California Los Angeles, Los Angeles, California, United States of America

[3] UCLA Department of Chemistry and Biochemistry, University of California Los Angeles, Los Angeles, California, United States of America

Knot statistics and comparison to the PDB

As discussed in the main text, for a knot to be well-defined, the two ends of the protein must be virtually closed[1,2], which sometimes leads to ambiguous results and requires additional visual inspection. Therefore, unfiltered results as presented here should thus be interpreted with caution. Both data banks (AlphaFold and PDB) also feature different degrees of

† These authors have contributed equally.
* Author to whom correspondence should be addressed: *virnau@uni-mainz.de*

redundancies, so the percentage of uniquely knotted proteins is lower. Without filtering, we find that the knotted proportion of the AlphaFold databank is about 2.26% which is a little larger than the proportion of knotted proteins in the PDB (approximately 1.3%, dividing the number of entries in KnotProt (ca. 2k)[3] by the number of entries in the PDB (ca. 150k)[4]). These values must be interpreted with caution as both databanks possess different kinds of redundancies. Moreover, knottiness increases with chain length and the average chain length in the AlphaFold databank is about 378 amino acids, whereas the average single-chain chain length in PDB is somewhat smaller at around 270 amino acids. For the above reasons, we consider the knotting probability to be encouragingly consistent with experimentally determined structures.

In Table 1, the number of knots in the AlphaFold databank in dependence of the knot type is listed. The knot Type is represented as N_i where N is the number of essential crossings and i enumerates different knots with the same number of essential crossings. The number of essential crossings is the minimum number of crossings in any projection of a certain knot to a plane. A higher number of essential crossings indicates a topologically more complex structure, wherein the complexity, e.g., the number of unique knots with the same number of essential crossings, depends exponentially on said number.

Knot type	Occurrences of this knot type
3_1	13557
4_1	2102
5_1	631
5_2	1319
6_1	241
6_2	264
6_3	136
7_1	35

7_2	74
7_3	43
7_4	58
7_5	75
7_6	77
7_7	40
$3_1 \# 3_1$	326
Other	2503

Table 1: Overview over the numbers of knots of a certain type found in the AlphaFold databank

Further topologically interesting proteins

Applying the filtering conditions discussed in the main text, our algorithm reveals 22 additional proteins with (allegedly) interesting topology: The prediction for protein A0A1C1CY86 (Glutathione S-transferase) contains a 5_2-knot. We note however, that a certain arc, which is substantial for the 5_2-knot, has very low (<50) per residue confidence. "Substantial for the knot" means here that small changes in the position of this arc may change the protein topology. PDBeFold[5,6] reveals that protein pdb:6hta:A is very likely a homologue with 55% sequence identity and 93% matching secondary structure. The latter protein is unknotted as it lacks this low-confidence arc and 18 residues at the N-terminus. AlphaFold's prediction for protein P9WHR4 (Carboxylesterase B) possesses a 5_1-knot. However, the N-terminus has very low per residue confidence and removal of the low-confidence region fully disentangles the protein. A similar case occurs for protein P65822 (putative hydrolase), protein A0A0R0HSA4 (uncharacterized) and protein X8FGD7 (hydrolase fold family). Protein P05845 (transposition protein) is reported to contain a 7_1-knot. We note however that an extended segment of the knotted core has very low per residue confidence and small changes to this segment reduce the 7_1-knot to a 5_1-knot. The AlphaFold prediction for protein P53336 (putative methyltransferase) contains a 5_1-knot, but slight variation of the N-terminus reduces the knot

from 5_1 to 3_1 which is in accordance with other observations for methyltransferases. Additionally, the prediction for carbonic anhydrase P24258 contains a 5_1-knot, which arises as the (low-confidence) N-terminus is threaded through an extended, unstructured low-confidence loop. Removal of the latter results in the well-known shallow trefoil knot structure of carbonic anhydrase. In the prediction for protein A0A077Z641 (vesicular GABA transporter), the N-terminus is buried in the structure such that the knot type depends heavily on the choice of closure. Our method resulted in a 5_1-knot. The prediction for protein Q57UX6 (uncharacterized) contains a 6_1-knot, which arises as the low confidence N-terminus threads trough two narrow loops. We consider this predicted structure to be very unlikely. A special case is protein Q8DP63 (Peptidoglycan-N-acetylglucosamine deacetylase). There, standard knot detection algorithms may find a 5_1-knot, which is however an artifact due to the employed closure method as the center of mass of the protein is located far away from the presumably knotted region. A similar issue also occurs for methyltransferase X8F3T9 which is reported to contain a $3_1\#3_1$-composite knot but only contains a single 3_1-knot and for protein U7PSZ9 (uncharacterized) which is reported as a 5_2-knot that is actually just a 3_1-knot. Finally, we want to acknowledge that we found the 6_3-knot in von Willebrand factor A (identifiers O00534 and Q99KC8), which was also reported in Ref.[7], even though it does not satisfy the conditions stated above due to its length of above 780 aa.

Accuracy test using ERRAT

As discussed in the main text, for the composite knot Q4D5S2 (and its relatives), the ERRAT[8,9] program flags a beta strand segment around residues 100-110 as likely to be structurally incorrect (Fig. S1). Notably, the passage of the chain in this region is important for the knotted

topology. While the AlphaFold program assigns a high degree of confidence to the predicted structure in this region, our independent assessment emphasizes the need for confirmatory experimental studies.

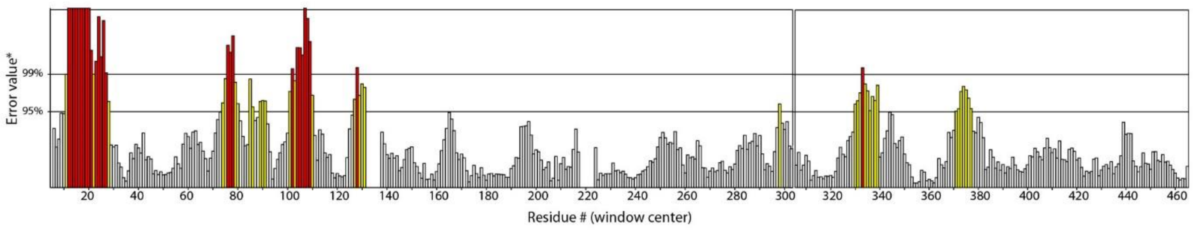

Figure S1: Analysis of model quality for protein Q4D5S2 using the program ERRAT[8,9]. The problematic region identified at the N-terminus is a reflection of the extended and disordered nature of the model there. The region near residues 100-110 appears problematic according to ERRAT though its confidence is reported as high by AlphaFold.

Per-residue confidence of discussed proteins

Supplementary figures S2-S6 depict the proteins from the figures in the main text as well as an illustration of the per-residue confidence scores by AlphaFold[10–12]. For those structures, no arcs which are substantial for the knots are of low confidence. While the link between the two substructures containing trefoil knots in Fig. S4 possesses low confidence, its exact position does not impact the overall protein topology as long as the two substructures are linked.

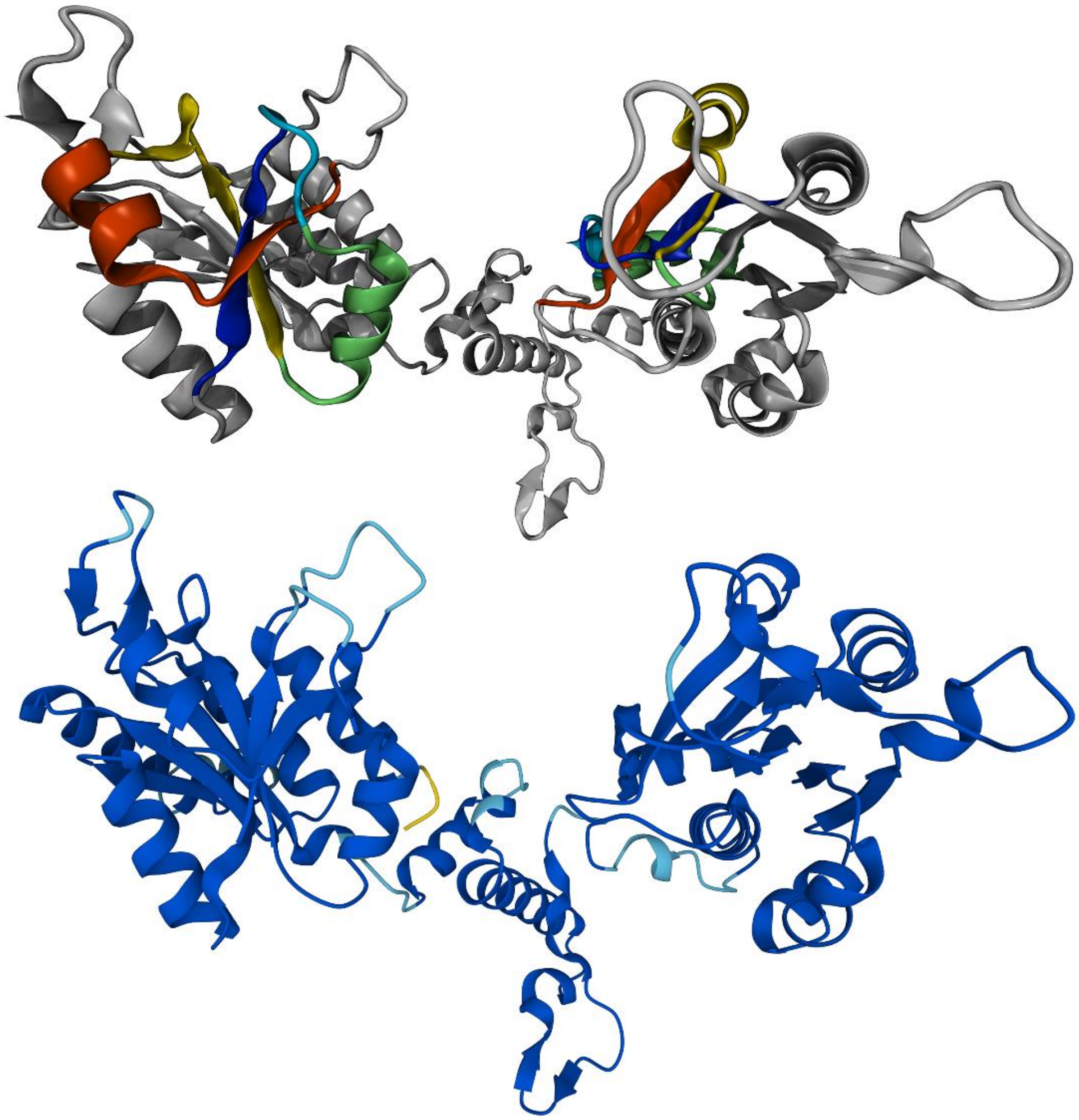

Figure S2: 3D structure of protein Q313J9 (methyltransferase) with two different color codings: Top: segments are colored as in the reduced representation in the main text. Bottom: segments are colored according to their per-residue confidence-score: dark blue 90 to 100, light blue 70 to 90, yellow 50 to 70, orange 0 to 50. The lower figure is generated by AlphaFold[10–12].

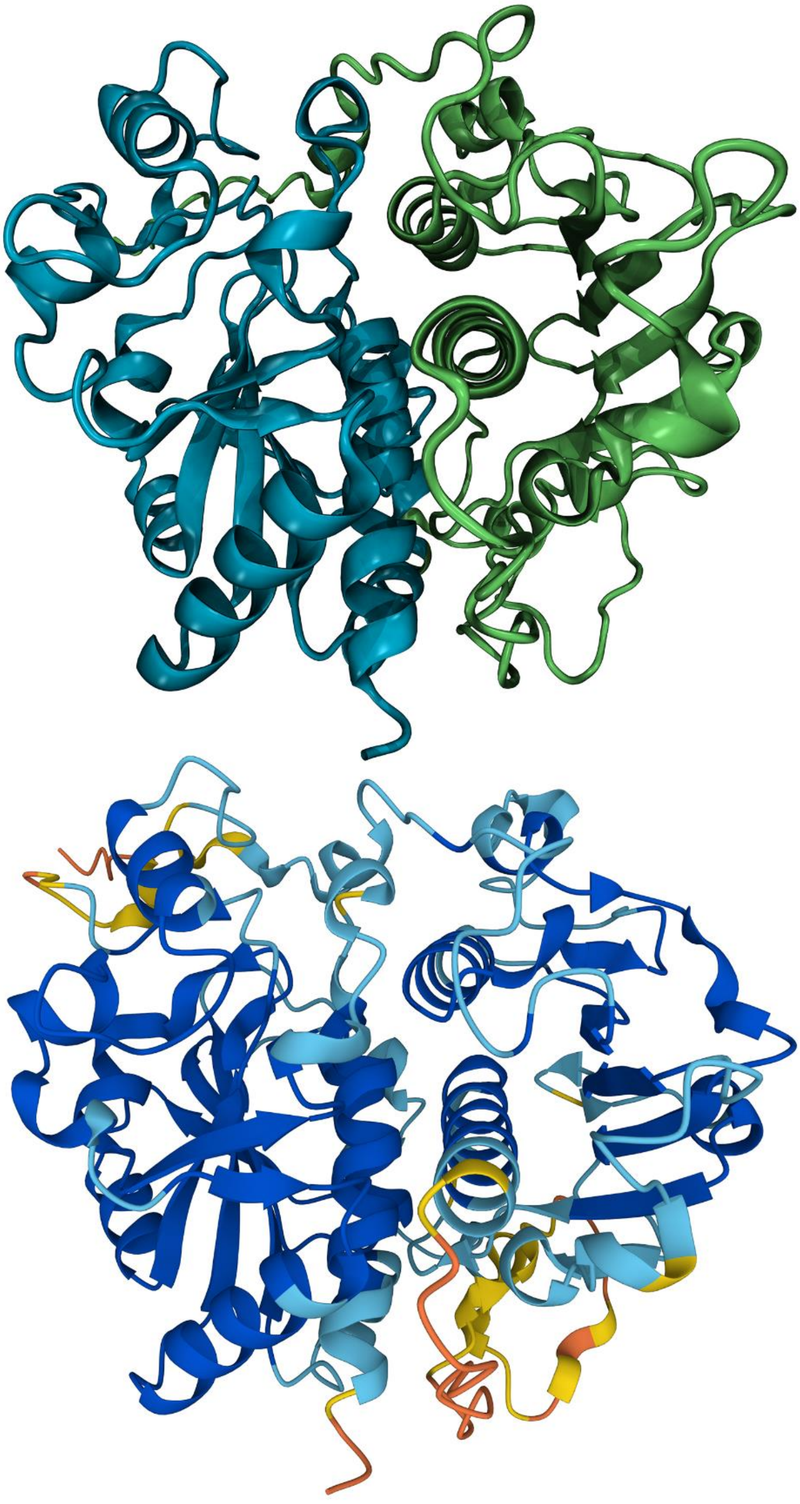

Figure S3: 3D structure of protein A4I142 (methyltransferase) with two different color codings: Top: segments are colored as in the reduced representation in the main text. Bottom: segments are colored according to their per-residue confidence-score: dark blue 90 to 100, light blue 70 to 90, yellow 50 to 70, orange 0 to 50. The lower figure is generated by AlphaFold[10–12].

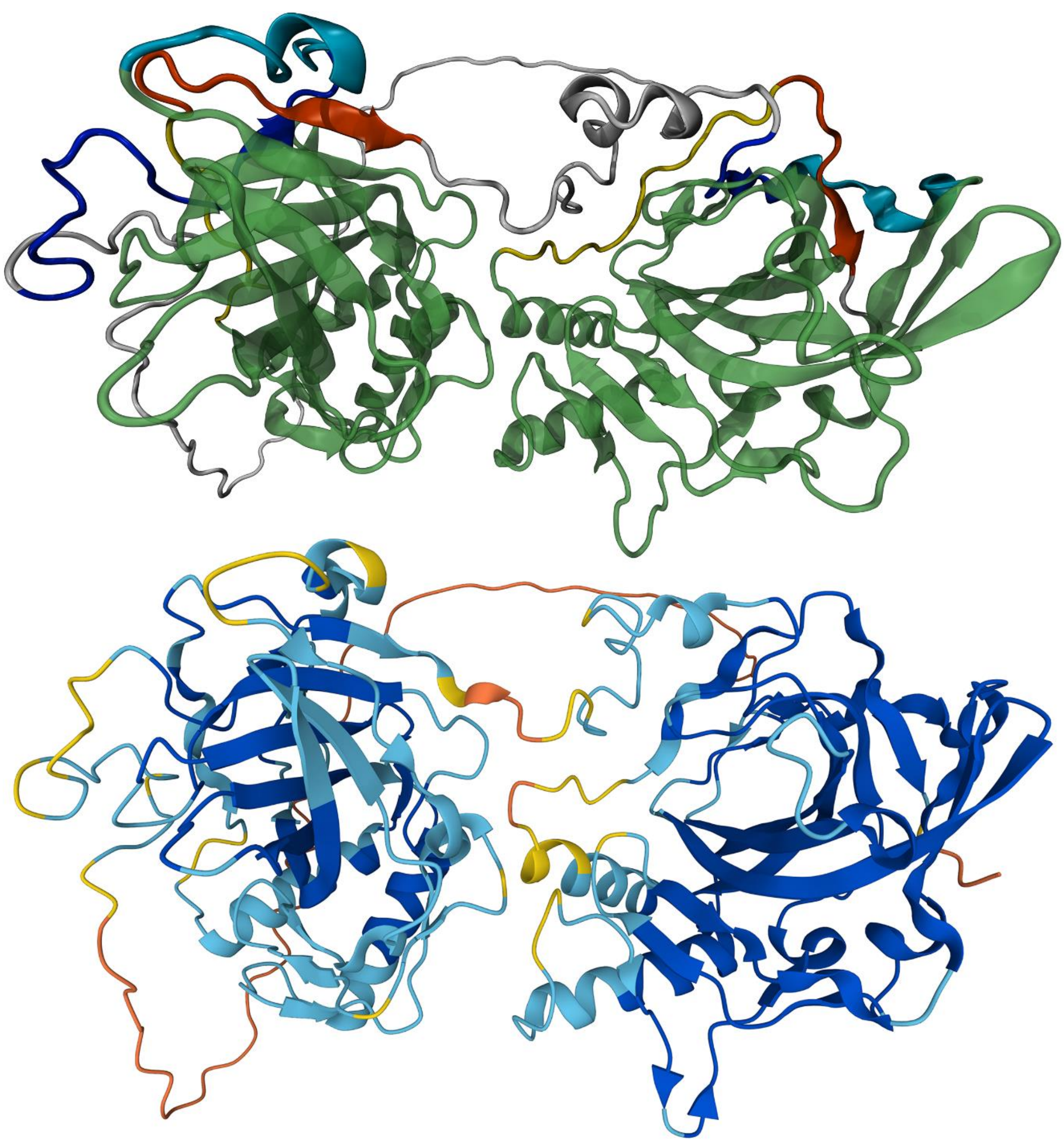

Figure S4: 3D structure of protein P54212 (carbonic anhydrase) with two different color codings: Top: segments are colored as in the reduced representation in the main text. Bottom: segments are colored according to their per-residue confidence-score: dark blue 90 to 100, light blue 70 to 90, yellow 50 to 70, orange 0 to 50. The lower figure is generated by AlphaFold[10–12].

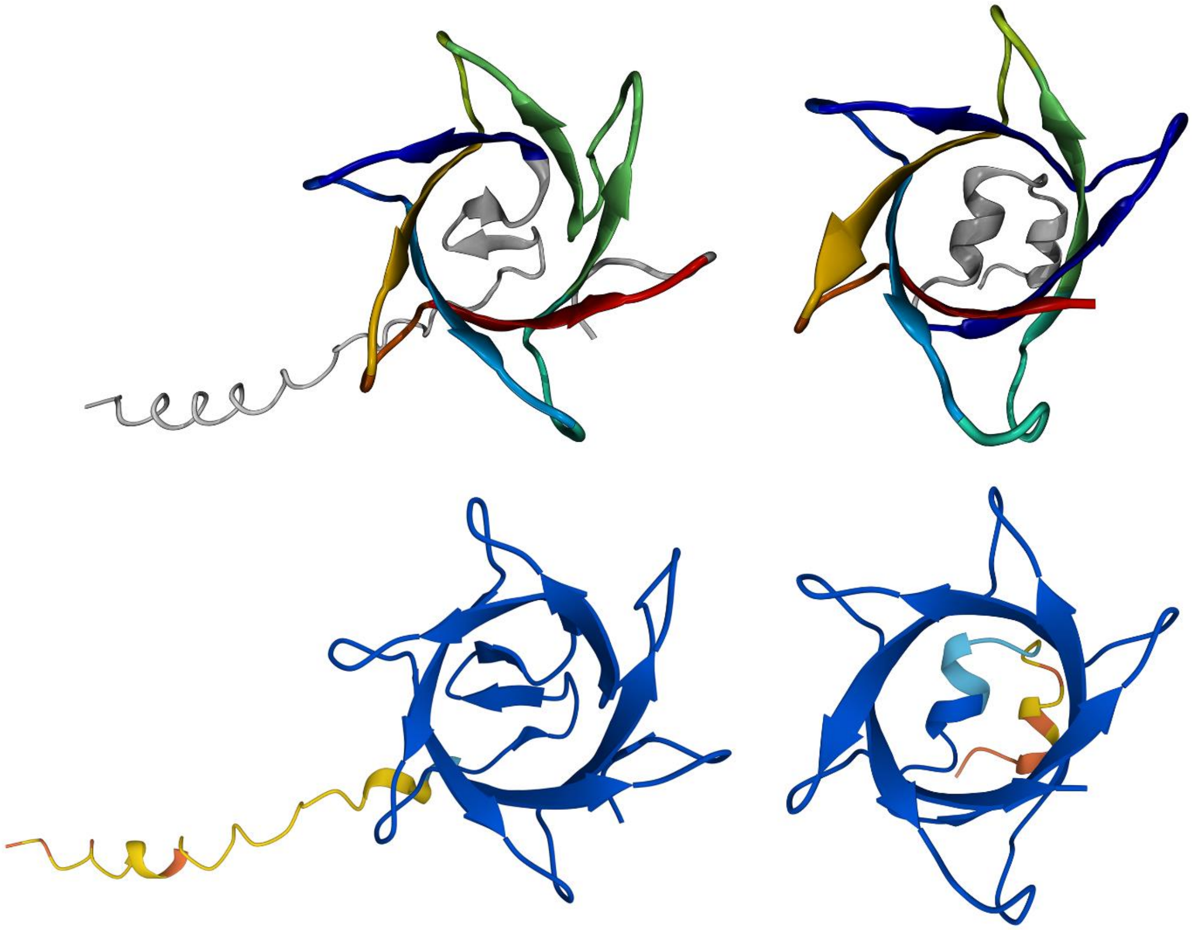

Figure S5: 3D structure of proteins P73136 (left) and Q9PR55 (right) with two different color codings: Top: segments are colored as in the reduced representation in the main text. Bottom: segments are colored according to their per-residue confidence-score: dark blue 90 to 100, light blue 70 to 90, yellow 50 to 70, orange 0 to 50. The lower figure is generated by AlphaFold[10–12].

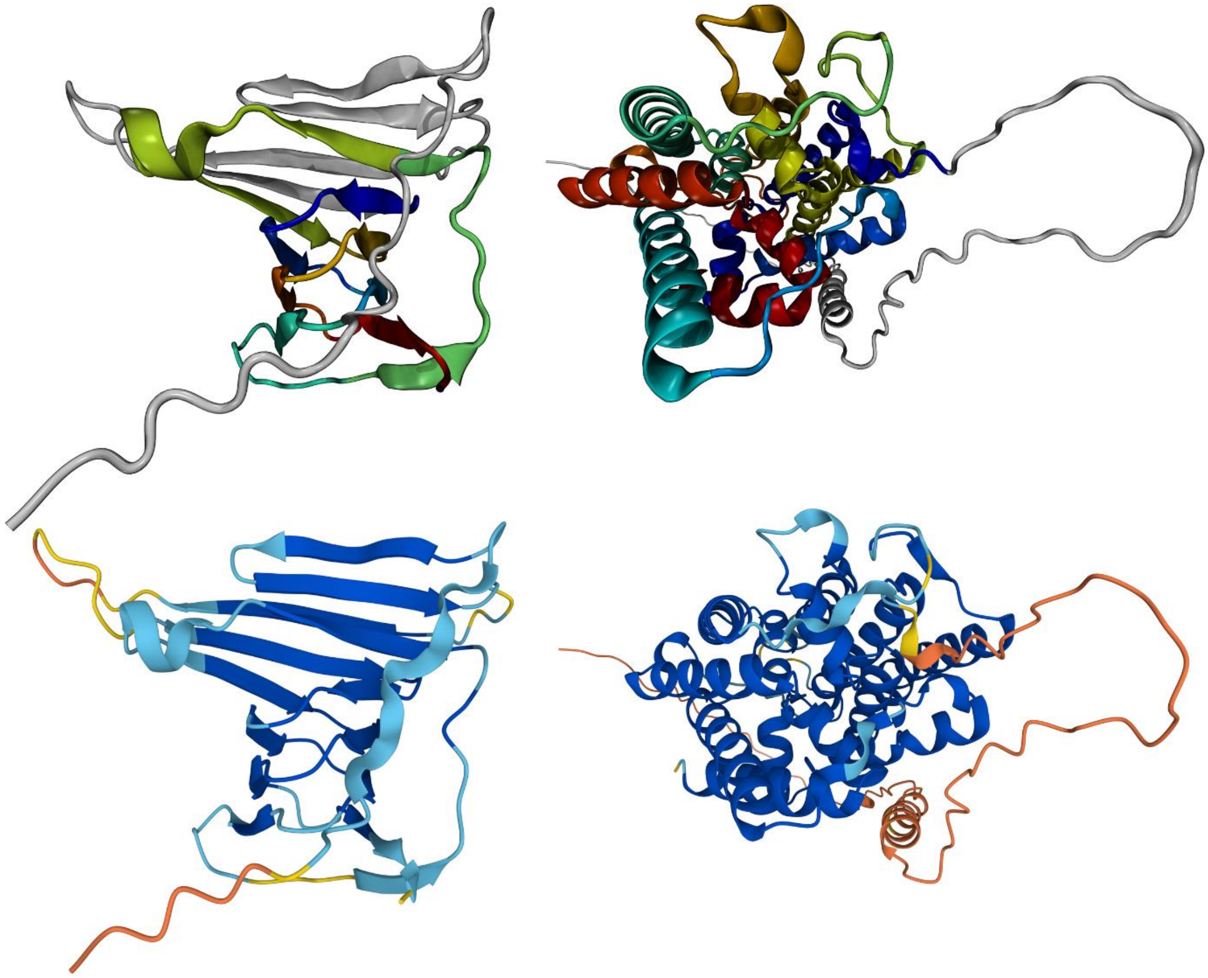

Figure S6: 3D structure of proteins A0A0K0IQS9 (left) and C1GYM9 (right) with two different color codings: Top: segments are colored as in the reduced representation in the main text. Bottom: segments are colored according to their per-residue confidence-score: dark blue 90 to 100, light blue 70 to 90, yellow 50 to 70, orange 0 to 50. The lower figure is generated by AlphaFold[10–12].

Structure alignment and sequence identity of $3_1\#3_1$-methyltransferases

We separate the methyltransferases containing composite knots into two categories: Those in which the two main segments containing the trefoil knots appear flexibly connected Q313J9 and Q72DU3, and those which preserve the presumed original dimer structure more strictly A4I142, Q4DMW6, Q4D5S2, Q4CYG6, Q4D7N4 and Q381U1. The two proteins in the first category appear very similar with 71.1 % sequence identity according to PDBeFold[5,6] and the secondary structure element alignment in Fig. S7. The single unmatched helix element in the middle is just a very short section which can be seen in Fig. S2 (bottom) in the righthand substructure in light blue. For the proteins in the second category, the sequence identity from PDBeFold is depicted in Fig. S8 and shows strong variations in the sequency identity between the different methyltransferases. The secondary structure element alignment is given in Fig. S9. In both secondary structure element alignment plots (Figs. S7 and S9) the region which may be roughly interpreted as connecting the two substructures is indicated. We observe that the secondary structure in Fig. S9 is preserved more strongly in the second

substructure, which may indicate that during the possible evolutional generation of the composite knots from unconnected dimers, the first substructure was altered more strongly. In Fig. S10 we provide the detailed, sequence-based structural alignment.

Structure Alignment

```
AF-Q72DU3-F1-model_v2.pdb:A SHHSHHSHSHSHhHHHHSHSHHSHSHSHSH
AF-Q313J9-F1-model_v2.pdb:A SHHSHHSHSHSHhHHHHSHSHHSHSHSHSH
```

Figure S7: Secondary structure element alignment for methyltransferases Q313J9 and Q72DU3 according to PDBeFold[5,6]. A violet rectangle roughly indicates the region connecting the two substructures.

Sequence Identity

	structure:	1	2	3	4	5	6
1	AF-A4I142-F1-model_v2.pdb:A		0.227	0.209	0.534	0.209	0.549
2	AF-Q381U1-F1-model_v2.pdb:A	0.227		0.648	0.275	0.645	0.278
3	AF-Q4CYG6-F1-model_v2.pdb:A	0.209	0.648		0.248	0.982	0.254
4	AF-Q4D5S2-F1-model_v2.pdb:A	0.534	0.275	0.248		0.248	0.910
5	AF-Q4D7N4-F1-model_v2.pdb:A	0.209	0.645	0.982	0.248		0.254
6	AF-Q4DMW6-F1-model_v2.pdb:A	0.549	0.278	0.254	0.910	0.254	

Figure S8: Sequence identity matrix for methyltransferases A4I142, Q4DMW6, Q4D5S2, Q4CYG6, Q4D7N4 and Q381U1 according to PDBeFold[5,6].

Secondary Structure Alignment

```
AF-A4I142-F1-model_v2.pdb:A   hsHh---hSshShSHh---HSHShHshShhhhHSHShhH-
AF-Q381U1-F1-model_v2.pdb:A   hsHhh--hSs-ShSHhh-hHSHS-Hh-Shh-sHSHSh-Hh
AF-Q4CYG6-F1-model_v2.pdb:A   hsHhhhhhSs-ShSHhh-hHSHS-Hh-Sh--sHSHSh-Hh
AF-Q4D5S2-F1-model_v2.pdb:A   hsHh----Sh-ShSHh---HSHShHshShhhsHSHSh-H-
AF-Q4D7N4-F1-model_v2.pdb:A   hsHhh--hSs-ShSHhhhhHSHShHshSh--sHSHS--Hh
AF-Q4DMW6-F1-model_v2.pdb:A   hsHh----SshShSHh---HSHShHshShhhsHSHSh-H-
```

Figure S9: Secondary structure element alignment for methyltransferases A4I142, Q4DMW6, Q4D5S2, Q4CYG6, Q4D7N4 and Q381U1 according to PDBeFold[5,6]. A violet rectangle roughly indicates the region connecting the two substructures.

3D Structural alignment

AF-A4I142-F1-model_v2.pdb:A		AF-Q381U1-F1-model_v2.pdb:A		AF-Q4CYG6-F1-model_v2.pdb:A		AF-Q4D5S2-F1-model_v2.pdb:A		AF-Q4D7N4-F1-model_v2.pdb:A		AF-Q4DMW6-F1-model_v2.pdb:A
		MET 1		MET 1				MET 1		
		PHE 2		MET 2				MET 2		
		PHE 3		PHE 3				PHE 3		
		ASN 4		SER 4				SER 4		
		GLN 5		SER 5				SER 5		
		LEU 6		ARG 6				ARG 6		
		LEU 7		LEU 7				LEU 7		
		LEU 8		LEU 8				LEU 8		
		ARG 9		HIS 9				HIS 9		
		LEU 10		LEU 10				LEU 10		
		ARG 11		ARG 11				ARG 11		
		VAL 12		ARG 12				ARG 12		
		THR 13		THR 13				THR 13		
		PRO 14		VAL 14				VAL 14		
		ARG 15		ARG 15				ARG 15		
MET 1		ARG 16		GLY 16				GLU 16		
PHE 2		PHE 17		PHE 17		MET 1		PHE 17		MET 1
LEU 3		PRO 18		ARG 18		LYS 2		ARG 18		LYS 2
HIS 4		GLN 19		GLN 19		TRP 3		GLN 19		TRP 3
SER 5		ALA 20		ALA 20		SER 4		ALA 20		SER 4
LEU 6		SER 21		SER 21		ALA 5		SER 21		ALA 5
LYS 7		ALA 22		ALA 22		VAL 6		ALA 22		VAL 6
ARG 8		PRO 23		PRO 23		CYS 7		PRO 23		CYS 7
LEU 9		PRO 24		PRO 24		LEU 8		PRO 24		LEU 8
ALA 10		ARG 25		ARG 25		LEU 9		ARG 25		LEU 9
HIS 11	|||	LYS 26	|||	GLY 26	|||	LYS 10	|||	GLY 26	|||	LYS 10
		VAL 27		PHE 27		ARG 11		PHE 27		
						ASP 12				
LYS 12	|||	CYS 28	|||	LEU 28	|||	PHE 13	|||	LEU 28	|||	ARG 11
		THR 29		ARG 29				ARG 29		ASP 12
		ASP 30		ASP 30				ASP 30		

H	ASP 13	|||	H	ASP 31	|||	H	ASP 31	|||		ASP 14	|||	H	ASP 31	|||		PHE 13
H	PHE 14		H	PRO 32		H	PRO 32					H	PRO 32		H	ASP 14
H	ASP 15		H	LEU 33		H	LEU 33					H	LEU 33			
			H	THR 34		H	THR 34					H	THR 34			
H	ALA 16		H	ARG 35		H	ARG 35					H	ARG 35		H	ALA 15
H	TRP 17	|||	H	TRP 36	|||	H	TRP 36	|||		ALA 15	|||	H	TRP 36	|||	H	TRP 16
H	GLY 18	|||	H	SER 37	|||	H	SER 37	|||		TRP 16	|||	H	SER 37	|||	H	ALA 17
										ALA 17						
H	TYR 19	|||	H	GLN 38	|||	H	GLU 38	|||		TYR 18	|||	H	GLU 38	|||	H	TYR 18
	HIS 20															SER 19
	ILE 21	|||	H	MET 39	|||	H	ARG 39	|||		SER 19	|||	H	ARG 39	|||		ILE 20
	GLN 22	|||	H	LEU 40	|||		LEU 40	|||		ILE 20	|||		LEU 40	|||		ASN 21
	TRP 23		H	ARG 41			LEU 41			ASN 21			LEU 41			
			H	LEU 42			ASN 42						ASN 42			
			H	GLY 43			GLY 43			TRP 22			GLY 43			
	PRO 24	|||		TRP 44	|||		TRP 44	|||		PRO 23	|||		TRP 44	|||		TRP 22
	GLN 25	|||		PRO 45	|||		PRO 45	|||		GLN 24	|||		PRO 45	|||		PRO 23
	ASN 26															
	GLN 27	|||		LYS 46	|||		ASN 46	|||	H	ARG 25	|||		ASN 46	|||		GLN 24
									H	LYS 26						ARG 25
																LYS 26
																SER 27
									H	SER 27						LEU 28
	ILE 28	|||		HIS 47	|||		ARG 47	|||	H	HIS 28	|||		ARG 47	|||		LEU 29
	LEU 29			CYS 48			CYS 48		H	LEU 29			CYS 48			
	SER 30	|||		HIS 49	|||		LEU 49	|||		PRO 30	|||		LEU 49	|||		PRO 30
	PRO 31															
	LEU 32															
	THR 33	|||		LEU 50	|||		HIS 50	|||		LEU 31	|||		HIS 50	|||		LEU 31
	GLY 34	|||		PRO 51	|||		SER 51	|||		LEU 32	|||		SER 51	|||		LEU 32
										GLY 33						GLY 33
	LYS 35	|||	S	HIS 52	|||	S	HIS 52	|||		SER 34	|||		HIS 52	|||		SER 34
S	VAL 36	|||	S	LEU 53	|||	S	LEU 53	|||	S	VAL 35	|||	S	LEU 53	|||	S	VAL 35
S	ASN 37	|||	S	SER 54	|||	S	PHE 54	|||	S	SER 36	|||	S	PHE 54	|||	S	SER 36

S	PHE 38	|||	S	VAL 55	|||	S	VAL 55	|||	S	LEU 37	|||	S	VAL 55	|||	S	LEU 37
S	VAL 39	|||	S	GLY 56	|||	S	GLY 56	|||	S	THR 38	|||	S	GLY 56	|||	S	THR 38
S	VAL 40	|||	S	LEU 57	|||	S	LEU 57	|||	S	VAL 39	|||	S	LEU 57	|||	S	VAL 39
	GLU 41	|||		GLU 58	|||		GLU 58	|||		GLU 40	|||		GLU 58	|||		GLU 40
	ASP 42	|||		GLY 59	|||		GLY 59	|||		ASP 41	|||		GLY 59	|||		ASP 41
	ILE 43	|||		CYS 60	|||		CYS 60	|||		ILE 42	|||		CYS 60	|||		ILE 42
	LYS 44	|||		THR 61	|||		THR 61	|||		LYS 43	|||		THR 61	|||		LYS 43
H	PHE 45	|||	H	SER 62	|||	H	SER 62	|||	H	PHE 44	|||	H	SER 62	|||	H	PHE 44
H	ALA 46	|||	H	ALA 63	|||	H	ALA 63	|||	H	ALA 45	|||	H	ALA 63	|||	H	ALA 45
H	TYR 47	|||	H	PHE 64	|||	H	PHE 64	|||	H	TYR 46	|||	H	PHE 64	|||	H	TYR 46
H	ASN 48	|||	H	ASN 65	|||	H	ASN 65	|||	H	ASN 47	|||	H	ASN 65	|||	H	ASN 47
H	ALA 49	|||	H	ALA 66	|||	H	ALA 66	|||	H	ALA 48	|||	H	ALA 66	|||	H	ALA 48
H	TRP 50	|||	H	MET 67	|||	H	MET 67	|||	H	TRP 49	|||	H	MET 67	|||	H	TRP 49
H	SER 51	|||	H	ASN 68	|||	H	ASN 68	|||	H	CYS 50	|||	H	ASN 68	|||	H	CYS 50
H	LEU 52	|||	H	SER 69	|||	H	SER 69	|||	H	LEU 51	|||	H	SER 69	|||	H	LEU 51
H	LEU 53	|||	H	ILE 70	|||	H	ILE 70	|||	H	LEU 52	|||	H	ILE 70	|||	H	LEU 52
H	ARG 54	|||	H	ARG 71	|||	H	ARG 71	|||	H	ARG 53	|||	H	ARG 71	|||	H	ARG 53
H	LEU 55	|||	H	THR 72	|||	H	THR 72	|||	H	LEU 54	|||	H	THR 72	|||	H	LEU 54
H	ALA 56	|||	H	CYS 73	|||	H	CYS 73	|||	H	ALA 55	|||	H	CYS 73	|||	H	ALA 55
H	MET 57	|||	H	MET 74	|||	H	MET 74	|||	H	MET 56	|||	H	MET 74	|||	H	MET 56
H	PHE 58	|||	H	PHE 75	|||	H	HIS 75	|||	H	PHE 57	|||	H	HIS 75	|||	H	PHE 57
H	PHE 59	|||	H	TYR 76	|||	H	PHE 76	|||	H	PHE 58	|||	H	PHE 76	|||	H	PHE 58
	GLY 60	|||	H	GLY 77	|||		GLY 77	|||	H	GLY 59	|||		GLY 77	|||	H	GLY 59
	VAL 61	|||		THR 78	|||		THR 78	|||		THR 60	|||		THR 78	|||		THR 60
	GLU 62	|||		ALA 79	|||		THR 79	|||		GLY 61	|||		THR 79	|||		GLY 61
				VAL 80			PHE 80						PHE 80			
	HIS 63	|||		PRO 81	|||		PRO 81	|||		HIS 62	|||		PRO 81	|||		HIS 62
	PRO 64	|||		PRO 82	|||		PRO 82	|||		PRO 63	|||		PRO 82	|||		PRO 63
	LYS 65	|||		SER 83	|||		PHE 83	|||		LYS 64	|||		PHE 83	|||		LYS 64
	VAL 66	|||		PHE 84	|||		PHE 84	|||		ILE 65	|||		PHE 84	|||		ILE 65
	LEU 67	|||		LEU 85	|||		LEU 85	|||		LEU 66	|||		LEU 85	|||		LEU 66
	SER 68	|||		SER 86	|||		SER 86	|||		SER 67	|||		SER 86	|||		SER 67
	ASP 69	|||		LEU 87	|||		LEU 87	|||		PRO 68	|||		LEU 87	|||		PRO 68

	MET 70	|||		SER 88	|||		LYS 88	|||		LEU 69	|||		LYS 88	|||		LEU 69
	THR 71		H	LYS 89		H	ARG 89			VAL 70		H	ARG 89			VAL 70
			H	ALA 90		H	ALA 90					H	ALA 90			
			H	GLU 91		H	GLU 91			ASN 71		H	GLU 91			
	LYS 72	|||	H	ASP 92	|||	H	ASP 92	|||		THR 72	|||	H	ASP 92	|||		ASN 71
										THR 73						
	THR 73	|||	H	ILE 93	|||	H	ILE 93	|||		PRO 74	|||	H	ILE 93	|||		THR 72
	THR 74	|||		LYS 94	|||		ARG 94	|||		SER 75	|||		HIS 94	|||		THR 73
	PRO 75	|||		ASN 95	|||		SER 95	|||		MET 76	|||		SER 95	|||		PRO 74
	SER 76	|||		ILE 96	|||		LEU 96	|||		ASP 77	|||		LEU 96	|||		SER 75
	MET 77															MET 76
H	ASP 78															ASP 77
H	ILE 79															
H	MET 80															
H	LEU 81															ILE 78
H	SER 82															ILE 79
	GLY 83	|||		ARG 97	|||		HIS 97	|||		ILE 78	|||		HIS 97	|||		TYR 80
										MET 79						
H	ASN 84	|||		ASP 98	|||		PRO 98	|||		TYR 80	|||		PRO 98	|||		GLY 81
				ARG 99			ARG 99		H	GLY 81			ARG 99			GLY 82
				GLN 100			CYS 100		H	GLY 82			CYS 100			
				CYS 101			THR 101						THR 101			
				GLY 102			ALA 102						ALA 102			
				THR 103			GLY 103						ARG 103			
				ASP 104			LYS 104						LYS 104			
				ASP 105			THR 105						THR 105			
				ALA 106			ASN 106						ASN 106			
				GLY 107		H	SER 107						SER 107			
				SER 108		H	GLU 108						GLU 108			
				ASN 109		H	ASP 109						ASP 109			
				PRO 110		H	LEU 110						LEU 110			
				GLU 111		H	THR 111						THR 111			
			H	THR 112		H	ARG 112					H	ARG 112			
			H	THR 113		H	PRO 113					H	PRO 113			

			H	SER 114		H	GLY 114		H	ASN 83		H	GLY 114		H	ASN 83
H	ARG 85	|||	H	LEU 115	|||	H	ALA 115	|||	H	ARG 84	|||	H	ALA 115	|||	H	ARG 84
H	LEU 86	|||	H	VAL 116	|||	H	THR 116	|||	H	LEU 85	|||	H	THR 116	|||	H	LEU 85
H	PHE 87	|||	H	LEU 117	|||	H	LEU 117	|||	H	PHE 86	|||	H	LEU 117	|||	H	PHE 86
H	PHE 88	|||	H	ASN 118	|||	H	SER 118	|||	H	TYR 87	|||	H	SER 118	|||	H	TYR 87
H	MET 89	|||		ARG 119	|||		GLN 119	|||	H	MET 88	|||		GLN 119	|||	H	MET 88
H	HIS 90	|||		VAL 120	|||		VAL 120	|||	H	HIS 89	|||		VAL 120	|||	H	HIS 89
	SER 91	|||		ALA 121	|||		ALA 121	|||	H	ALA 90	|||		ALA 121	|||		ALA 90
	ARG 92	|||		GLN 122	|||		ARG 122	|||		GLU 91	|||		ARG 122	|||		GLU 91
	VAL 93	|||		PHE 123	|||		PHE 123	|||		VAL 92	|||		PHE 123	|||		VAL 92
	LYS 94	|||		GLY 124	|||		GLY 124	|||		GLY 93	|||		GLY 124	|||		GLY 93
			H	LYS 125		H	GLU 125					H	GLU 125			
			H	GLU 126		H	ASP 126					H	ASP 126			
	ALA 95	|||	H	ALA 127	|||	H	LEU 127	|||		ALA 94	|||	H	LEU 127	|||		ALA 94
	ASN 96	|||	H	PHE 128	|||	H	PHE 128	|||		SER 95	|||	H	PHE 128	|||		SER 95
	LEU 97	|||	H	PHE 129	|||	H	LEU 129	|||		LEU 96	|||	H	LEU 129	|||		LEU 96
			H	ARG 130		H	ASP 130					H	ASP 130			
				GLY 131		H	ASP 131					H	ASP 131			
						H	PRO 132					H	PRO 132			
				GLN 132		H	SER 133					H	SER 133			
	SER 98	|||		TRP 133	|||	H	PHE 134	|||		SER 97	|||	H	PHE 134	|||		SER 97
				ALA 134												
	ARG 99	|||		GLU 135	|||	H	GLY 135	|||		ARG 98	|||	H	GLY 135	|||		ARG 98
	SER 100	|||		ASN 136	|||		GLN 136	|||		HIS 99	|||		GLN 136	|||		TYR 99
	LYS 101	|||		PHE 137	|||		ILE 137	|||		LYS 100	|||		ILE 137	|||		LYS 100
S	VAL 102	|||		PRO 138	|||		PRO 138	|||		PRO 101	|||		PRO 138	|||		PRO 101
S	ARG 103	|||	S	LEU 139	|||	S	LEU 139	|||	S	LYS 102	|||	S	LEU 139	|||	S	LYS 102
S	ILE 104	|||	S	VAL 140	|||	S	VAL 140	|||	S	VAL 103	|||	S	VAL 140	|||	S	VAL 103
S	ALA 105	|||	S	ALA 141	|||	S	ALA 141	|||	S	ALA 104	|||	S	ALA 141	|||	S	ALA 104
S	LEU 106	|||	S	LEU 142	|||	S	LEU 142	|||	S	LEU 105	|||	S	LEU 142	|||	S	LEU 105
	THR 107	|||		GLU 143	|||		GLU 143	|||		THR 106	|||		GLU 143	|||		THR 106
	PRO 108	|||		ASN 144	|||		ASN 144	|||		PRO 107	|||		ASN 144	|||		PRO 107
	ASN 109	|||		PHE 145	|||		TYR 145	|||		SER 108	|||		TYR 145	|||		SER 108

	HIS 110	|||		THR 146	|||		THR 146	|||		HIS 109	|||		THR 146	|||		HIS 109
	PRO 111	|||		HIS 147	|||		ASN 147	|||		PRO 110	|||		ASN 147	|||		PRO 110
	ARG 112	|||		ARG 148	|||		ARG 148	|||		ASP 111	|||		ARG 148	|||		ASP 111
S	ALA 113	|||	S	SER 149	|||	S	SER 149	|||		ALA 112	|||	S	SER 149	|||	S	ALA 112
S	PHE 114	|||	S	GLN 150	|||	S	GLU 150	|||		GLN 113	|||	S	GLU 150	|||	S	GLN 113
S	PRO 115	|||	S	SER 151	|||	S	SER 151	|||		PRO 114	|||	S	SER 151	|||	S	PRO 114
	VAL 116	|||		LEU 152	|||		ILE 152	|||		VAL 115	|||		ILE 152	|||		VAL 115
	GLN 117	|||		PHE 153	|||		LEU 153	|||		GLN 116	|||		LEU 153	|||		GLN 116
	GLU 118	|||		THR 154	|||		SER 154	|||		SER 117	|||		SER 154	|||		SER 117
	LEU 119	|||		CYS 155	|||		CYS 155	|||		PHE 118	|||		CYS 155	|||		PHE 118
H	VAL 120	|||		ARG 156	|||		ARG 156	|||	H	PRO 119	|||		ARG 156	|||	H	PRO 119
H	TRP 121	|||		ILE 157	|||		LEU 157	|||	H	TRP 120	|||		LEU 157	|||	H	TRP 120
H	ASN 122								H	ARG 121					H	ARG 121
H	THR 123								H	LYS 122					H	LYS 122
H	GLN 124								H	VAL 123					H	VAL 123
H	PHE 125	|||		SER 158	|||		SER 158	|||	H	ILE 124	|||		SER 158	|||	H	ILE 124
	ARG 126			SER 159			SER 159		H	ARG 125			SER 159		H	ARG 125
	PHE 127								H	GLU 126					H	GLU 126
	ARG 128								H	ALA 127					H	ALA 127
	ASP 129								H	GLY 128					H	GLY 128
									H	GLU 129					H	GLU 129
									H	ARG 130					H	ARG 130
										ARG 131						ARG 131
										THR 132						THR 132
										GLY 133						ASP 133
										ASN 134						ASN 134
										GLU 135						GLU 135
	HIS 130									ASP 136						ASP 136
	GLN 131									CYS 137						CYS 137
	SER 132									ASP 138						ASP 138
	ALA 133									GLY 139						GLY 139

	GLY 134	|||	S	VAL 160	|||	S	SER 160	|||		GLY 140	|||		PRO 160	|||		GLY 140
S	VAL 135	|||	S	ALA 161	|||	S	ALA 161	|||	S	VAL 141	|||	S	ALA 161	|||	S	VAL 141
S	GLU 136	|||	S	ARG 162	|||	S	ARG 162	|||	S	GLU 142	|||	S	ARG 162	|||	S	GLU 142
S	LEU 137	|||	S	LEU 163	|||	S	ILE 163	|||	S	ILE 143	|||	S	ILE 163	|||	S	ILE 143
S	VAL 138	|||	S	LEU 164	|||	S	VAL 164	|||	S	VAL 144	|||	S	VAL 164	|||	S	VAL 144
S	LEU 139	|||	S	ILE 165	|||	S	VAL 165	|||	S	LEU 145	|||	S	VAL 165	|||	S	LEU 145
	GLY 140	|||		GLY 166	|||		GLY 166	|||		GLY 146	|||		GLY 166	|||		GLY 146
	MET 141	|||		HIS 167	|||		HIS 167	|||		MET 147	|||		HIS 167	|||		MET 147
	GLU 142	|||		GLU 168	|||		GLU 168	|||		GLU 148	|||		GLU 168	|||		GLU 148
				ASN 169			ASN 169						ASN 169			
	ASN 143	|||		ARG 170	|||		ASN 170	|||		ASN 149	|||		ASN 170	|||		ASN 149
	GLY 144	|||		GLY 171	|||		GLY 171	|||		GLY 150	|||		GLY 171	|||		GLY 150
	LEU 145	|||		VAL 172	|||		VAL 172	|||		LEU 151	|||		VAL 172	|||		LEU 151
H	SER 146	|||	H	SER 173	|||	H	SER 173	|||	H	SER 152	|||	H	SER 173	|||	H	SER 152
H	GLN 147	|||	H	SER 174	|||	H	ARG 174	|||	H	GLU 153	|||	H	ARG 174	|||	H	GLU 153
H	GLU 148	|||	H	LYS 175	|||	H	LYS 175	|||	H	ASN 154	|||	H	LYS 175	|||	H	ASN 154
H	VAL 149	|||	H	TYR 176	|||	H	TYR 176	|||	H	VAL 155	|||	H	TYR 176	|||	H	VAL 155
H	ALA 150	|||	H	ILE 177	|||	H	ILE 177	|||	H	VAL 156	|||	H	ILE 177	|||	H	VAL 156
H	ASP 151	|||		GLU 178	|||	H	ASP 178	|||	H	SER 157	|||	H	ASP 178	|||	H	SER 157
				CYS 179			LYS 179						LYS 179			
				ASP 180			MET 180						MET 180			
				ASN 181			ASP 181						ASP 181			
				ALA 182			VAL 182						GLU 182			
				THR 183			SER 183						SER 183			
				ILE 184												
				SER 185												
				ASP 186												
				GLU 187			GLY 184						GLY 184			
				SER 188			GLY 185						ARG 185			
				ASP 189			LYS 186						LYS 186			
				GLY 190			GLY 187						GLY 187			
				GLN 191			CYS 188						CYS 188			
H	ALA 152	|||		CYS 192	|||		ARG 189	|||		GLN 158	|||		ARG 189	|||		GLN 158

H	CYS 153	|||		ALA 193	|||		ALA 190	|||		CYS 159	|||		ALA 190	|||		CYS 159
	ASN 154	|||		ASP 194	|||		GLU 191	|||		ASP 160	|||		GLU 191	|||		ASP 160
S	LEU 155	|||	S	CYS 195	|||	S	ARG 192	|||	S	TYR 161	|||	S	ARG 192	|||	S	TYR 161
S	CYS 156	|||	S	VAL 196	|||	S	VAL 193	|||	S	CYS 162	|||	S	VAL 193	|||	S	CYS 162
S	THR 157	|||	S	VAL 197	|||	S	VAL 194	|||	S	LEU 163	|||	S	VAL 194	|||	S	LEU 163
S	TYR 158	|||	S	TYR 198	|||	S	TYR 195	|||	S	TYR 164	|||	S	TYR 195	|||	S	TYR 164
	ILE 159	|||		VAL 199	|||		VAL 196	|||		ILE 165	|||		VAL 196	|||		ILE 165
	PRO 160	|||		PRO 200	|||		PRO 197	|||		PRO 166	|||		PRO 197	|||		PRO 166
	GLN 161	|||		GLN 201	|||		GLN 198	|||		GLN 167	|||		GLN 198	|||		GLN 167
	TYR 162	|||		TYR 202	|||		TYR 199	|||		TYR 168	|||		TYR 199	|||		TYR 168
	GLY 163	|||		GLY 203	|||		GLY 200	|||		GLY 169	|||		GLY 200	|||		GLY 169
	SER 164	|||		THR 204	|||		THR 201	|||		SER 170	|||		THR 201	|||		SER 170
	VAL 165	|||		ILE 205	|||		ILE 202	|||		ILE 171	|||		ILE 202	|||		ILE 171
	GLY 166	|||		SER 206	|||		SER 203	|||		GLY 172	|||		SER 203	|||		GLY 172
	SER 167	|||		SER 207	|||		SER 204	|||		SER 173	|||		SER 204	|||		SER 173
	LEU 168	|||		LEU 208	|||		LEU 205	|||		LEU 174	|||		LEU 205	|||		LEU 174
H	SER 169	|||	H	ASN 209	|||	H	ASN 206	|||	H	SER 175	|||	H	ASN 206	|||	H	SER 175
H	MET 170	|||	H	VAL 210	|||	H	VAL 207	|||	H	MET 176	|||	H	VAL 207	|||	H	MET 176
H	LEU 171	|||	H	VAL 211	|||	H	VAL 208	|||	H	ILE 177	|||	H	VAL 208	|||	H	ILE 177
H	SER 172	|||	H	THR 212	|||	H	THR 209	|||	H	SER 178	|||	H	THR 209	|||	H	SER 178
H	ALA 173	|||	H	SER 213	|||	H	SER 210	|||	H	ALA 179	|||	H	SER 210	|||	H	ALA 179
H	LEU 174	|||	H	MET 214	|||	H	MET 211	|||	H	MET 180	|||	H	MET 211	|||	H	MET 180
H	ALA 175	|||	H	GLY 215	|||	H	GLY 212	|||	H	ALA 181	|||	H	GLY 212	|||	H	ALA 181
H	ILE 176	|||	H	ILE 216	|||	H	ILE 213	|||	H	ILE 182	|||	H	ILE 213	|||	H	ILE 182
H	ALA 177	|||	H	ALA 217	|||	H	ALA 214	|||	H	ALA 183	|||	H	ALA 214	|||	H	ALA 183
H	ALA 178	|||	H	LEU 218	|||	H	LEU 215	|||	H	LEU 184	|||	H	LEU 215	|||	H	LEU 184
H	HIS 179	|||	H	PHE 219	|||	H	PHE 216	|||	H	HIS 185	|||	H	PHE 216	|||	H	HIS 185
H	SER 180	|||	H	TYR 220	|||	H	TYR 217	|||	H	SER 186	|||	H	TYR 217	|||	H	SER 186
H	THR 181	|||	H	ALA 221	|||	H	ALA 218	|||	H	ALA 187	|||	H	ALA 218	|||	H	ALA 187
H	TRP 182	|||	H	PHE 222	|||	H	THR 219	|||	H	ALA 188	|||	H	THR 219	|||	H	ALA 188
H	ARG 183	|||	H	LEU 223	|||	H	LEU 220	|||	H	SER 189	|||	H	LEU 220	|||	H	SER 189
H	ALA 184	|||	H	ASP 224	|||	H	ASP 221	|||	H	ALA 190	|||	H	ASP 221	|||	H	ALA 190
H	ALA 185	|||	H	GLN 225	|||	H	ALA 222	|||	H	HIS 191	|||	H	ALA 222	|||	H	HIS 191
H	VAL 186	|||	H	ASN 226	|||	H	ARG 223	|||	H	VAL 192	|||	H	ARG 223	|||	H	VAL 192

H	ARG 187	|||	H	PHE 227	|||	H	TYR 224	|||	H	ASP 193	|||	H	TYR 224	|||	H	GLU 193
H	GLU 188	|||		PRO 228	|||		PRO 225	|||	H	GLN 194	|||		PRO 225	|||	H	GLN 194
H	GLU 189									TYR 195					H	CYS 195
H	GLY 190															
	ASN 191															
	THR 192															
	SER 193									MET 196						MET 196
	ASP 194	|||		HIS 229	|||		SER 226	|||		ARG 197	|||		SER 226	|||		ARG 197
	THR 195			SER 230			ALA 227			ASP 198			ALA 227			ASP 198
										ASP 199						ASP 199
										ASP 200						ASP 200
										ARG 201						ARG 201
										VAL 202						VAL 202
	SER 196	|||		ARG 231	|||		ARG 228	|||		SER 203	|||		ARG 228	|||		SER 203
	LEU 197									PRO 204						PRO 204
	ALA 198	|||		SER 232	|||		SER 229	|||		CYS 205	|||		THR 229	|||		CYS 205
				LEU 233		H	LEU 230						LEU 230			
	PRO 199	|||		MET 234	|||	H	LEU 231	|||		SER 206	|||		LEU 231	|||		SER 206
	HIS 200			THR 235		H	ARG 232			SER 207			ARG 232			SER 207
	ARG 201			GLU 236		H	ASP 233			ARG 208			ASP 233			ARG 208
	ALA 202		H	THR 237		H	THR 234			GLY 209		H	THR 234			GLY 209
	LEU 203		H	THR 238		H	GLU 235			HIS 210		H	LYS 235			HIS 210
	HIS 204		H	LYS 239			ASP 236			MET 211		H	ASP 236			MET 211
	GLY 205		H	ARG 240			GLY 237			PRO 212		H	GLY 237			PRO 212
	HIS 206		H	ALA 241			ARG 238			LEU 213		H	ARG 238			LEU 213
	MET 207			PHE 242			GLU 239			SER 214			GLU 239			SER 214
	PRO 208			CYS 243			ASN 240			ASN 215			ASN 240			ASN 215
	LEU 209			PRO 244			GLY 241			GLY 216			GLY 241			GLY 216
	SER 210			ASN 245		H	SER 242			SER 217			ARG 242			SER 217
	ASN 211		H	THR 246		H	GLY 243					H	GLY 243			
			H	ASP 247		H	HIS 244					H	HIS 244			
			H	ASP 248		H	GLU 245					H	GLU 245			
			H	GLU 249		H	LEU 246					H	LEU 246			

			H	LEU 250		H	ASP 247					H	ASP 247			
			H	GLU 251		H	GLU 248					H	GLU 248			
			H	ALA 252		H	LEU 249					H	LEU 249			
			H	LEU 253		H	HIS 250					H	HIS 250			
			H	LEU 254		H	ALA 251					H	ALA 251			
			H	SER 255		H	TYR 252					H	TYR 252			
			H	TYR 256		H	GLN 253					H	GLN 253			
			H	GLN 257		H	ARG 254					H	ARG 254			
			H	ARG 258												
			H	CYS 259		H	PHE 255					H	PHE 255			
				PHE 260			PHE 256						PHE 256			
				ARG 261			ARG 257						ARG 257			
				GLU 262			GLN 258						GLN 258			
				ARG 263			GLN 259						GLN 259			
				ILE 264			ILE 260						ILE 260			
				PRO 265			PRO 261						PRO 261			
				THR 266			ARG 262						ARG 262			
				THR 267			THR 263					H	THR 263			
				HIS 268			ALA 264					H	ALA 264			
				GLY 269			ALA 265					H	ALA 265			
				TYR 270			GLU 266					H	GLU 266			
	SER 212			GLY 271			CYS 267			LEU 218		H	CYS 267			LEU 218
	PRO 213			VAL 272			ASN 268			LYS 219			ASN 268			LYS 219
	ASP 214			PRO 273			SER 269			VAL 220			SER 269			VAL 220
	ARG 215			SER 274			SER 270			THR 221			SER 270			THR 221
	GLN 216			ARG 275			ARG 271			LYS 222			ARG 271			LYS 222
	ARG 217			ILE 276			VAL 272			GLY 223			VAL 272			GLY 223
	ASP 218			ASP 277			ASP 273			ASN 224			ASP 273			ASP 224
	SER 219			PRO 278			GLN 274			ASN 225			GLN 274			ASN 225
	LEU 220			ARG 279			ARG 275			LEU 226			ARG 275			LEU 226
	PRO 221			PRO 280			PRO 276			PRO 227			PRO 276			PRO 227
H	HIS 222			ILE 281			ILE 277			HIS 228			ILE 277			HIS 228
H	GLU 223	|||	H	HIS 282	|||	H	HIS 278	|||	H	GLU 229	|||	H	HIS 278	|||	H	GLU 229
H	MET 224	|||	H	PRO 283	|||	H	PRO 279	|||	H	THR 230	|||	H	PRO 279	|||	H	THR 230

H	ASP 225	|||	H	LEU 284	|||	H	LEU 280	|||	H	ASN 231	|||	H	LEU 280	|||	H	ASN 231
H	LEU 226	|||	H	TYR 285	|||	H	TYR 281	|||	H	LEU 232	|||	H	TYR 281	|||	H	LEU 232
H	LEU 227	|||	H	PHE 286	|||	H	TYR 282	|||	H	LEU 233	|||	H	TYR 282	|||	H	LEU 233
				GLY 287			LYS 283			SER 234			LYS 283			SER 234
H	ALA 228	|||		LYS 288	|||		LYS 284	|||		LEU 235	|||		LYS 284	|||		LEU 235
	CYS 229															
H	SER 230	|||	H	ASN 289	|||	H	ASP 285	|||	H	SER 236	|||	H	ASP 285	|||	H	SER 236
H	ASN 231	|||	H	VAL 290	|||	H	ALA 286	|||	H	ASN 237	|||	H	ALA 286	|||	H	ASN 237
H	ALA 232	|||	H	GLU 291	|||	H	THR 287	|||	H	VAL 238	|||	H	THR 287	|||	H	ALA 238
H	ALA 233	|||	H	ASN 292	|||	H	GLY 288	|||	H	GLU 239	|||	H	GLY 288	|||	H	GLU 239
H	ILE 234	|||	H	ILE 293	|||	H	ILE 289	|||	H	ILE 240	|||	H	ILE 289	|||	H	ILE 240
H	LYS 235	|||	H	ILE 294	|||	H	LEU 290	|||	H	ALA 241	|||	H	LEU 290	|||	H	ALA 241
H	ASP 236	|||	H	ASP 295	|||	H	GLU 291	|||	H	GLU 242	|||	H	GLU 291	|||	H	GLU 242
H	LEU 237	|||	H	THR 296	|||	H	MET 292	|||	H	ILE 243	|||	H	MET 292	|||	H	ILE 243
H	LEU 238	|||	H	HIS 297	|||	H	HIS 293	|||	H	LEU 244	|||	H	HIS 293	|||	H	LEU 244
H	GLU 239	|||	H	ASN 298	|||	H	ARG 294	|||	H	ARG 245	|||	H	ARG 294	|||	H	ARG 245
H	GLU 240	|||	H	HIS 299	|||	H	HIS 295	|||	H	ALA 246	|||	H	HIS 295	|||	H	ALA 246
H	ARG 241	|||	H	LEU 300	|||	H	LEU 296	|||	H	ARG 247	|||	H	LEU 296	|||	H	ARG 247
H	ARG 242	|||	H	ARG 301	|||	H	ARG 297	|||	H	ARG 248	|||	H	ARG 297	|||	H	ARG 248
H	ARG 243	|||	H	LYS 302	|||	H	GLN 298	|||	H	MET 249	|||	H	GLN 298	|||	H	MET 249
	ALA 244	|||	H	LEU 303	|||	H	LEU 299	|||	H	SER 250	|||	H	LEU 299	|||		SER 250
	TYR 245	|||	H	LEU 304	|||	H	MET 300	|||	H	TYR 251	|||	H	MET 300	|||		TYR 251
	ASP 246	|||	H	LEU 305	|||	H	LEU 301	|||		PRO 252	|||	H	LEU 301	|||		PRO 252
			H	ARG 306		H	ARG 302					H	ARG 302			
			H	ALA 307		H	VAL 303					H	VAL 303			
			H	CYS 308		H	CYS 304					H	CYS 304			
				SER 309		H	SER 305						SER 305			
				ASP 310			HIS 306						HIS 306			
				GLN 311			ARG 307						GLY 307			
				ARG 312			ARG 308						ARG 308			
				ARG 313			PHE 309						PHE 309			
				PRO 314			SER 310						SER 310			
				GLY 315			SER 311						SER 311			

				LYS 316			SER 312						SER 312			
				GLY 317			PRO 313						THR 313			
				ARG 318			GLY 314						GLY 314			
				CYS 319			ARG 315						ARG 315			
	LEU 247	|||		PHE 320	|||		PHE 316	|||		MET 253	|||		PHE 316	|||		MET 253
S	GLN 248	|||		GLY 321	|||		GLY 317	|||	S	GLN 254	|||		GLY 317	|||	S	GLN 254
S	VAL 249	|||	S	LEU 322	|||	S	LEU 318	|||	S	LEU 255	|||	S	LEU 318	|||	S	LEU 255
S	SER 250	|||	S	SER 323	|||	S	PHE 319	|||	S	SER 256	|||	S	PHE 319	|||	S	SER 256
S	VAL 251	|||	S	VAL 324	|||	S	VAL 320	|||	S	VAL 257	|||	S	VAL 320	|||	S	VAL 257
S	LEU 252	|||	S	LEU 325	|||	S	VAL 321	|||	S	MET 258	|||	S	VAL 321	|||	S	MET 258
S	ILE 253	|||	S	CYS 326	|||	S	TYR 322	|||	S	VAL 259	|||	S	TYR 322	|||	S	VAL 259
S	TYR 254	|||	S	GLN 327	|||	S	GLU 323	|||	S	TYR 260	|||	S	GLU 323	|||	S	TYR 260
S	ASN 255	|||	S	ASN 328	|||	S	ASN 324	|||	S	ASN 261	|||	S	ASN 324	|||	S	ASN 261
	GLU 256	|||		GLU 329	|||		GLU 325	|||		GLU 262	|||		GLU 325	|||		GLU 262
	LEU 257	|||		ILE 330	|||		ARG 326	|||		PHE 263	|||		ARG 326	|||		PHE 263
																GLY 264
	GLY 258	|||	H	ASP 331	|||	H	ASP 327	|||		GLY 264	|||	H	ASP 327	|||		ASP 265
	ASP 259		H	LEU 332		H	GLN 328			ASP 265		H	GLN 328			
	ARG 260	|||	H	ARG 333	|||	H	ARG 329	|||		ARG 266	|||	H	ARG 329	|||		ARG 266
H	ASN 261	|||	H	ASN 334	|||	H	SER 330	|||	H	ASN 267	|||	H	SER 330	|||	H	ASN 267
H	ILE 262	|||	H	LEU 335	|||	H	PHE 331	|||	H	ILE 268	|||	H	PHE 331	|||	H	ILE 268
H	GLY 263	|||	H	GLY 336	|||	H	ALA 332	|||	H	GLY 269	|||	H	ALA 332	|||	H	GLY 269
H	ALA 264	|||	H	GLY 337	|||	H	GLY 333	|||	H	ALA 270	|||	H	GLY 333	|||	H	ALA 270
H	VAL 265	|||	H	ILE 338	|||	H	ILE 334	|||	H	ILE 271	|||	H	ILE 334	|||	H	ILE 271
H	ILE 266	|||	H	VAL 339	|||	H	VAL 335	|||	H	MET 272	|||	H	VAL 335	|||	H	MET 272
H	ARG 267	|||	H	ARG 340	|||	H	ARG 336	|||	H	ARG 273	|||	H	ARG 336	|||	H	ARG 273
H	ASN 268	|||	H	SER 341	|||	H	ASN 337	|||	H	ASN 274	|||	H	ASN 337	|||	H	ASN 274
H	ALA 269	|||	H	ALA 342	|||	H	ALA 338	|||	H	ALA 275	|||	H	ALA 338	|||	H	ALA 275
H	ASN 270	|||	H	ASN 343	|||	H	ASN 339	|||	H	ASN 276	|||	H	ASN 339	|||	H	ASN 276
H	VAL 271	|||	H	ALA 344	|||	H	ALA 340	|||	H	VAL 277	|||	H	ALA 340	|||	H	VAL 277
H	PHE 272	|||	H	PHE 345	|||	H	PHE 341	|||	H	PHE 278	|||	H	PHE 341	|||	H	PHE 278
	ASN 273	|||		LEU 346	|||		LEU 342	|||		ASN 279	|||		LEU 342	|||		ASN 279
	CYS 274	|||		VAL 347	|||		VAL 343	|||		CYS 280	|||		VAL 343	|||		CYS 280
	GLU 275	|||		ASP 348	|||		ASP 344	|||		GLU 281	|||		ASP 344	|||		GLU 281

	TYR 276	|||	S	ASN 349	|||	S	GLN 345	|||	S	GLN 282	|||	S	GLN 345	|||	S	GLN 282
S	VAL 277	|||	S	ILE 350	|||	S	VAL 346	|||	S	MET 283	|||	S	VAL 346	|||	S	MET 283
S	ALA 278	|||	S	PHE 351	|||	S	LEU 347	|||	S	ILE 284	|||	S	LEU 347	|||	S	ILE 284
S	ILE 279	|||	S	TYR 352	|||	S	TYR 348	|||	S	VAL 285	|||	S	TYR 348	|||	S	VAL 285
S	VAL 280	|||	S	PHE 353	|||	S	PHE 349	|||	S	LEU 286	|||	S	PHE 349	|||	S	LEU 286
S	ASN 281	|||	S	GLY 354	|||	S	GLY 350	|||	S	HIS 287	|||	S	GLY 350	|||	S	HIS 287
	ARG 282	|||		ARG 355	|||		ARG 351	|||		ARG 288	|||		ARG 351	|||		ARG 288
	ARG 283	|||		ARG 356	|||		LYS 352	|||		ARG 289	|||		LYS 352	|||		ARG 289
	ARG 284	|||		LYS 357	|||		LYS 353	|||		LYS 290	|||		LYS 353	|||		LYS 290
	PHE 285	|||		ILE 358	|||		ILE 354	|||		PHE 291	|||		ILE 354	|||		PHE 291
										ASN 292						ASN 292
H	ASN 286	|||		ASN 359	|||		ASN 355	|||	H	ARG 293	|||	H	ASN 355	|||	H	ARG 293
H	ARG 287			VAL 360			VAL 356					H	VAL 356			
H	ARG 288	|||		VAL 361	|||		VAL 357	|||	H	ARG 294	|||	H	VAL 357	|||	H	ARG 294
H	GLY 289	|||		GLY 362	|||		GLY 358	|||	H	GLY 295	|||	H	GLY 358	|||	H	GLY 295
H	ALA 290	|||		THR 363	|||		THR 359	|||	H	ALA 296	|||	H	THR 359	|||	H	ALA 296
H	LEU 291	|||		VAL 364	|||		VAL 360	|||	H	LEU 297	|||	H	VAL 360	|||	H	LEU 297
H	GLY 292	|||	H	GLY 365	|||	H	GLY 361	|||	H	GLY 298	|||	H	GLY 361	|||	H	GLY 298
H	THR 293	|||	H	THR 366	|||	H	THR 362	|||	H	THR 299	|||	H	THR 362	|||	H	THR 299
H	HIS 294	|||	H	GLN 367	|||	H	GLN 363	|||	H	GLN 300	|||	H	GLN 363	|||	H	GLN 300
H	LYS 295	|||	H	HIS 368	|||	H	HIS 364	|||	H	HIS 301	|||	H	HIS 364	|||	H	HIS 301
H	VAL 296	|||	H	TYR 369	|||	H	TYR 365	|||	H	LEU 302	|||	H	TYR 365	|||	H	LEU 302
	MET 297	|||		THR 370	|||		THR 366	|||		ILE 303	|||		THR 366	|||		ILE 303
	ASN 298	|||		PRO 371	|||		PRO 367	|||	S	LYS 304	|||		PRO 367	|||	S	ASN 304
S	VAL 299	|||		PRO 372	|||		PRO 368	|||	S	THR 305	|||		PRO 368	|||	S	THR 305
S	LEU 300	|||		THR 373	|||		VAL 369	|||	S	LEU 306	|||	S	VAL 369	|||	S	LEU 306
S	PHE 301	|||		TYR 374	|||		TYR 370	|||	S	PHE 307	|||	S	TYR 370	|||	S	PHE 307
S	PHE 302	|||		LEU 375	|||		LEU 371	|||	S	CYS 308	|||	S	LEU 371	|||	S	TYR 308
				GLY 376			GLY 372					S	GLY 372			
	GLU 303	|||		ASP 377	|||		PRO 373	|||		SER 309	|||		PRO 373	|||		SER 309
	THR 304	|||		LEU 378	|||		PHE 374	|||		GLY 310	|||		PHE 374	|||		GLY 310
	VAL 305			PHE 379			ASP 375			VAL 311			VAL 375			VAL 311
				GLU 380			VAL 376						VAL 376			

				GLY 381			GLU 377						GLU 377			
				CYS 382			ASP 378						ASP 378			
				GLY 383			GLY 379						GLY 379			
				SER 384												
			H	SER 385												
			H	ILE 386												
			H	GLU 387												
			H	ASP 388			SER 380						SER 380			
			H	MET 389		H	ASP 381					H	ASP 381			
			H	GLU 390		H	ALA 382					H	ALA 382			
			H	PRO 391		H	GLU 383					H	GLU 383			
	ASN 306		H	GLN 392		H	THR 384			ASP 312		H	THR 384			ASP 312
H	ASP 307	|||	H	LEU 393	|||	H	TRP 385	|||	H	ASP 313	|||	H	TRP 385	|||	H	ASP 313
						H	ALA 386					H	ALA 386			
						H	THR 387					H	THR 387			
H	ASP 308	|||	H	ASN 394	|||	H	LYS 388	|||	H	ALA 314	|||	H	LYS 388	|||	H	ALA 314
H	ALA 309		H	LEU 395					H	GLN 315					H	GLN 315
H	ALA 310	|||	H	MET 396	|||	H	LEU 389	|||	H	CYS 316	|||	H	LEU 389	|||	H	CYS 316
			H	LYS 397		H	LEU 390					H	LEU 390			
H	ARG 311	|||	H	LYS 398	|||	H	GLN 391	|||	H	GLN 317	|||	H	GLN 391	|||	H	GLN 317
H	GLN 312	|||	H	ARG 399	|||	H	ARG 392	|||	H	LYS 318	|||	H	ARG 392	|||	H	LYS 318
H	LEU 313	|||	H	LEU 400	|||	H	VAL 393	|||	H	SER 319	|||	H	VAL 393	|||	H	SER 319
H	PHE 314	|||	H	ASP 401	|||	H	GLU 394	|||	H	LEU 320	|||	H	GLU 394	|||	H	LEU 320
H	GLU 315	|||	H	GLU 402	|||	H	LEU 395	|||		GLU 321	|||	H	LEU 395	|||		GLU 321
H	GLY 316		H	ALA 403		H	GLU 396			GLY 322		H	GLU 396			GLY 322
S	TYR 317	|||		ASN 404	|||	H	ASN 397	|||	S	TYR 323	|||	H	ASN 397	|||	S	TYR 323
				VAL 405		H	GLY 398					H	GLY 398			
				PRO 406			GLY 399						GLY 399			
							ASN 400						ASN 400			
				CYS 407			ILE 401						ILE 401			
S	GLU 318	|||	S	ARG 408	|||	S	SER 402	|||	S	THR 324	|||	S	SER 402	|||	S	THR 324
S	VAL 319	|||	S	TRP 409	|||	S	TRP 403	|||	S	ILE 325	|||	S	LEU 403	|||	S	ILE 325
S	TRP 320	|||	S	TRP 410	|||	S	TRP 404	|||	S	TRP 326	|||	S	TRP 404	|||	S	TRP 326
S	LEU 321	|||	S	PHE 411	|||	S	PHE 405	|||	S	LEU 327	|||	S	PHE 405	|||	S	LEU 327

S	LEU 322	|||	S	LEU 412	|||	S	LEU 406	|||	S	LEU 328	|||	S	LEU 406	|||	S	LEU 328
	TYR 323	|||	S	ASP 413	|||	S	ASP 407	|||	S	TYR 329	|||	S	ASP 407	|||	S	TYR 329
	PRO 324	|||		CYS 414	|||		CYS 408	|||		GLN 330	|||		CYS 408	|||		GLN 330
	TYR 325	|||		GLY 415	|||		GLY 409	|||		TYR 331	|||		GLY 409	|||		TYR 331
	TYR 326	|||	H	HIS 416	|||		HIS 410	|||		TYR 332	|||		HIS 410	|||		TYR 332
			H	LYS 417			ALA 411			PRO 333			ALA 411			PRO 333
			H	PHE 418			PHE 412			TYR 334			PHE 412			
	PRO 327	|||	H	LEU 419	|||		LEU 413	|||		LEU 335	|||		LEU 413	|||		TYR 334
	ASN 328									LYS 336						
	LEU 329															
	VAL 330															
	MET 331	|||	H	TYR 420	|||		TYR 414	|||		ILE 337	|||		TYR 414	|||		LEU 335
	TYR 332		H	SER 421						TYR 338						LYS 336
	GLU 333															ILE 337
	ALA 334															TYR 338
	GLU 335															GLU 339
	ARG 336															ASP 340
	LYS 337															PRO 341
	GLY 338															ASN 342
H	ASP 339															
H	ALA 340															
H	SER 341															
H	ASN 342															
H	SER 343															
	SER 344															
	SER 345															
	THR 346															
	PHE 347															
	VAL 348															GLY 343
	ARG 349															ASP 344
	HIS 350															GLU 345
	GLU 351															ASP 346
H	ASP 352															ALA 347
H	PRO 353															THR 348

H	VAL 354		H	ALA 422						GLU 339						PHE 349
H	LEU 355	|||	H	SER 423	|||		MET 415	|||		ASP 340	|||		MET 415	|||		ILE 350
H	GLN 356		H	ARG 424			ASP 416			PRO 341			ASP 416			ARG 351
H	ALA 357		H	SER 425			SER 417			ASN 342			SER 417			PRO 352
H	TRP 358		H	ARG 426			THR 418			GLY 343			THR 418			GLU 353
H	GLY 359		H	VAL 427			SER 419			ASP 344			SER 419		H	SER 354
H	ALA 360		H	SER 428			VAL 420			GLU 345			VAL 420		H	SER 355
H	THR 361		H	ASP 429			ASN 421			ASP 346			ASN 421		H	LEU 356
	GLN 362		H	LEU 430			GLU 422			ALA 347			GLU 422		H	PHE 357
	HIS 363		H	GLN 431			THR 423			THR 348			THR 423		H	GLN 358
	GLU 364		H	ASP 432			ALA 424			PHE 349			ALA 424		H	GLU 359
				LEU 433		H	LYS 425			ILE 350			GLU 425		H	TRP 360
						H	GLU 426			ARG 351			LYS 426		H	LEU 361
										PRO 352					H	GLN 362
										GLU 353						
									H	SER 354						
									H	THR 355						
									H	LEU 356						
									H	LEU 357						
									H	GLN 358						
									H	GLU 359						
									H	TRP 360						
									H	LEU 361						
									H	GLN 362						
									H	GLY 363						
										SER 364					H	CYS 363
										TYR 365						SER 364
				GLN 434		H	PRO 427			GLY 366			PRO 427			HIS 365
	LEU 365			GLY 435		H	GLU 428			MET 367			GLU 428			GLY 366
	THR 366		H	SER 436		H	LYS 429			ASN 368			LYS 429			MET 367
	SER 367		H	GLY 437		H	MET 430			PRO 369			MET 430			ASN 368
	GLU 368		H	GLY 438		H	GLU 431			ASP 370			GLU 431			PRO 369
H	HIS 369		H	SER 439		H	THR 432		H	HIS 371			THR 432			ASP 370

H	PRO 370		H	GLN 440		H	ARG 433		H	PRO 372			ARG 433		H	HIS 371
H	LEU 371		H	ALA 441		H	ASP 434		H	MET 373			GLY 434		H	PRO 372
H	VAL 372		H	ALA 442		H	SER 435		H	MET 374		H	SER 435		H	MET 373
H	VAL 373		H	LEU 443		H	VAL 436		H	ASN 375		H	VAL 436		H	MET 374
H	ARG 374	|||	H	GLU 444	|||	H	GLU 437	|||	H	PHE 376	|||	H	GLU 437	|||	H	THR 375
			H	ALA 445		H	ALA 438		H	HIS 377		H	ALA 438		H	PHE 376
			H	VAL 446		H	LEU 439					H	LEU 439			
			H	LYS 447		H	THR 440					H	THR 440		H	HIS 377
H	TYR 375	|||	H	TRP 448	|||	H	TRP 441	|||	H	ALA 378	|||	H	TRP 441	|||	H	ALA 378
H	PRO 376		H	TYR 449		H	TYR 442		H	SER 379		H	TYR 442		H	SER 379
H	HIS 377		H	GLU 450		H	ASN 443		H	HIS 380		H	ASN 443		H	HIS 380
			H	THR 451		H	THR 444					H	THR 444			
			H	ARG 452		H	HIS 445					H	HIS 445			
			H	TYR 453		H	ARG 446					H	ARG 446			
			H	SER 454		H	ALA 447						ALA 447			
H	LEU 378			ASP 455			ASP 448		H	MET 381			ASP 448		H	LEU 381
H	CYS 379			PRO 456			PRO 449		H	ILE 382			PRO 449		H	ILE 382
H	GLN 380	|||		ASN 457	|||		ASP 450	|||		GLY 383	|||		ASP 450	|||		GLY 383
	LYS 381	|||		CYS 458	|||		PHE 451	|||		THR 384	|||		PHE 451	|||		THR 384
	ALA 382	|||	S	MET 459	|||	S	THR 452	|||	S	ASP 385	|||	S	THR 452	|||	S	ASP 385
	VAL 383	|||	S	LEU 460	|||	S	LEU 453	|||	S	VAL 386	|||	S	LEU 453	|||	S	VAL 386
	PHE 384	|||	S	SER 461	|||	S	SER 454	|||	S	TYR 387	|||	S	SER 454	|||	S	TYR 387
	LEU 385	|||		LEU 462	|||		LEU 455	|||		LEU 388	|||		LEU 455	|||		LEU 388
	ASP 386	|||		CYS 463	|||		CYS 456	|||		ASP 389	|||		CYS 456	|||		ASP 389
				GLU 464			ASP 457						ASP 457			
H	ASP 387	|||	H	SER 465	|||	H	THR 458	|||	H	ASP 390	|||	H	THR 458	|||	H	ASP 390
H	ASP 388	|||	H	GLU 466	|||	H	GLU 459	|||	H	SER 391	|||	H	GLU 459	|||	H	SER 391
H	LEU 389	|||	H	GLU 467	|||	H	GLU 460	|||	H	ASP 392	|||	H	GLU 460	|||	H	ASP 392
H	SER 390	|||	H	THR 468	|||	H	ARG 461	|||	H	SER 393	|||	H	ARG 461	|||	H	SER 393
H	LEU 391	|||	H	LEU 469	|||	H	ILE 462	|||	H	VAL 394	|||	H	ILE 462	|||	H	VAL 394
H	ALA 392	|||	H	ARG 470	|||	H	ARG 463	|||	H	HIS 395	|||	H	ARG 463	|||	H	HIS 395
H	SER 393	|||	H	GLU 471	|||	H	GLU 464	|||	H	ALA 396	|||	H	GLU 464	|||	H	ALA 396
H	CYS 394	|||	H	ALA 472	|||	H	THR 465	|||	H	ALA 397	|||	H	THR 465	|||	H	ALA 397
H	VAL 395	|||	H	ILE 473	|||	H	ALA 466	|||	H	VAL 398	|||	H	ALA 466	|||	H	VAL 398

H	GLN 396								H	ARG 399					H	GLN 399
H	ASP 397								H	HIS 400					H	HIS 400
H	VAL 398	|||		ARG 474	|||		GLN 467	|||	H	ALA 401	|||		GLN 467	|||	H	ALA 401
H	LYS 399								H	ALA 402					H	ALA 402
H	ARG 400								H	ASN 403					H	ASN 403
H	ARG 401								H	GLU 404					H	GLU 404
	LYS 402									GLY 405						GLY 405
	LEU 403	|||		GLU 475	|||		GLY 468	|||		TYR 406	|||		GLY 468	|||		TYR 406
	ARG 404									ARG 407						ARG 407
S	GLY 405	|||		GLY 476	|||		GLY 469	|||	S	GLY 408	|||		GLY 469	|||	S	GLY 408
S	ILE 406	|||	S	VAL 477	|||	S	VAL 470	|||	S	ILE 409	|||	S	VAL 470	|||	S	ILE 409
S	LEU 407	|||	S	LEU 478	|||	S	VAL 471	|||	S	VAL 410	|||	S	VAL 471	|||	S	VAL 410
S	LEU 408	|||	S	LEU 479	|||	S	LEU 472	|||	S	LEU 411	|||	S	LEU 472	|||	S	LEU 411
S	ALA 409	|||	S	VAL 480	|||	S	LEU 473	|||	S	VAL 412	|||	S	LEU 473	|||	S	VAL 412
S	VAL 410	|||	S	VAL 481	|||	S	VAL 474	|||	S	VAL 413	|||	S	VAL 474	|||	S	VAL 413
	PRO 411	|||		PRO 482	|||		PRO 475	|||		PRO 414	|||		PRO 475	|||		PRO 414
	GLU 412	|||		GLN 483	|||		GLN 476	|||		GLU 415	|||		GLN 476	|||		GLU 415
	GLU 413	|||		GLU 484	|||		GLU 477	|||		GLU 416	|||		GLU 477	|||		GLU 416
	GLY 414	|||		GLY 485	|||		GLY 478	|||		GLY 417	|||		GLY 478	|||		GLY 417
	CYS 415	|||		ILE 486	|||		ILE 479	|||		ALA 418	|||		ILE 479	|||		ALA 418
	SER 416	|||		LEU 487	|||		LEU 480	|||		SER 419	|||		LEU 480	|||		SER 419
	PRO 417	|||		PRO 488	|||		PRO 481	|||		PHE 420	|||		PRO 481	|||		PHE 420
H	HIS 418	|||	H	PRO 489	|||	H	THR 482	|||	H	GLN 421	|||	H	PRO 482	|||	H	GLN 421
H	PRO 419	|||	H	LEU 490	|||	H	PHE 483	|||	H	ARG 422	|||	H	PHE 483	|||	H	ARG 422
H	CYS 420	|||	H	HIS 491	|||	H	CYS 484	|||	H	ASP 423	|||	H	CYS 484	|||	H	GLU 423
H	LEU 421	|||	H	ILE 492	|||	H	LEU 485	|||	H	ILE 424	|||	H	LEU 485	|||	H	ILE 424
H	CYS 422	|||	H	LEU 493	|||	H	LEU 486	|||	H	VAL 425	|||	H	LEU 486	|||	H	VAL 425
H	LYS 423	|||	H	GLN 494	|||	H	GLN 487	|||	H	LYS 426	|||	H	GLN 487	|||	H	LYS 426
H	LEU 424	|||		ARG 495	|||		ARG 488	|||	H	LYS 427	|||		ARG 488	|||	H	LYS 427
H	SER 425	|||		CYS 496	|||		CYS 489	|||	H	ALA 428	|||		CYS 489	|||	H	ALA 428
	HIS 426	|||		GLU 497	|||		GLN 490	|||		ARG 429	|||		GLN 490	|||		ARG 429
S	ARG 427	|||	S	LYS 498	|||	S	LYS 491	|||	S	ARG 430	|||	S	LYS 491	|||	S	ARG 430
S	VAL 428	|||	S	ILE 499	|||	S	ILE 492	|||	S	ILE 431	|||	S	ILE 492	|||	S	ILE 431

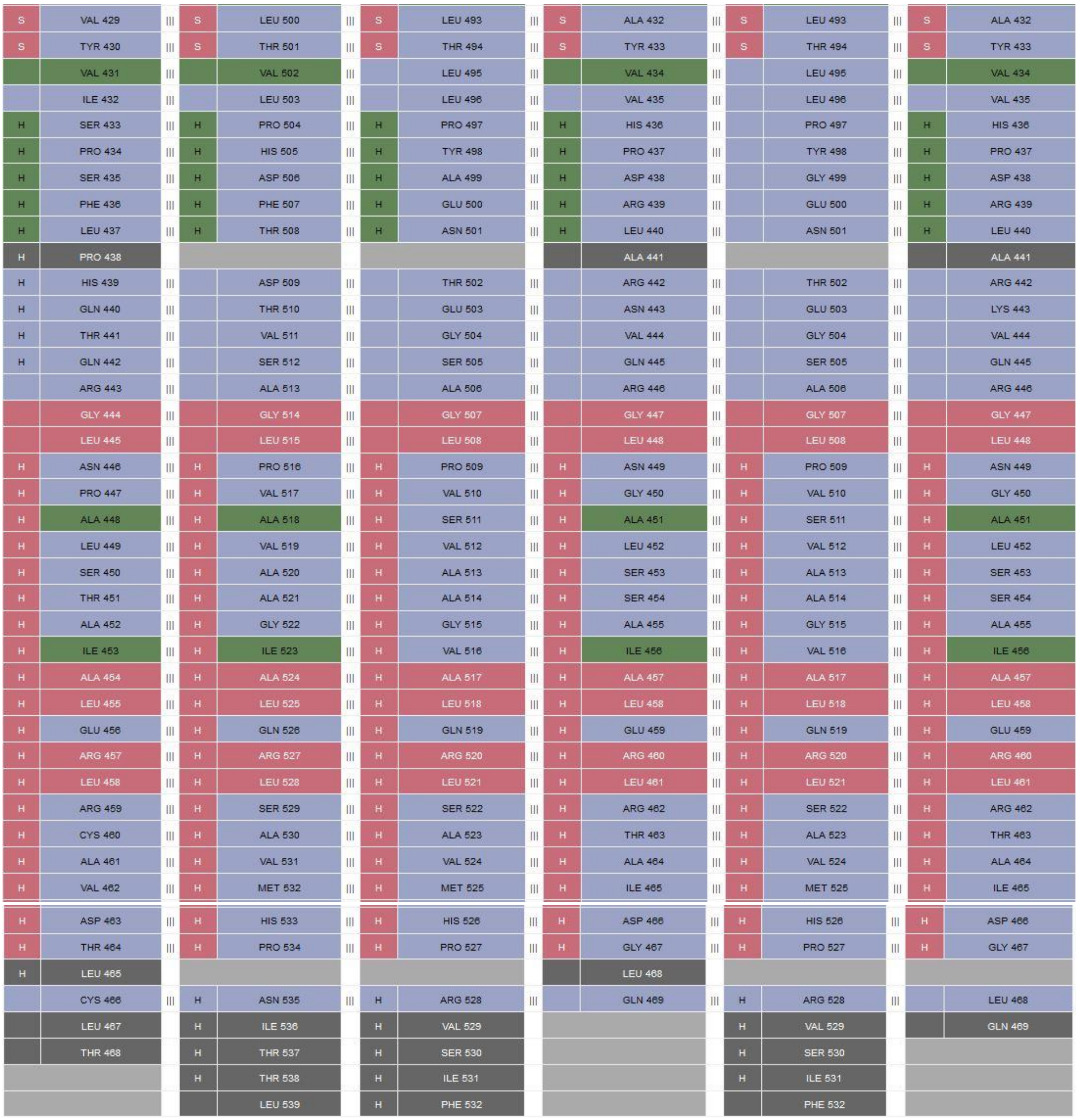

Figure S10: Detailed structural alignment for methyltransferases A4I142, Q4DMW6, Q4D5S2, Q4CYG6, Q4D7N4 and Q381U1 according to PDBeFold[5,6].

References

1. Virnau P (2010) Detection and visualization of physical knots in macromolecules. Physics Procedia 6:117–125.

2. Kolesov G, Virnau P, Kardar M, Mirny LA (2007) Protein knot server: detection of knots in protein structures. Nucleic Acids Res 35:W425-428.

3. Jamroz M, Niemyska W, Rawdon EJ, Stasiak A, Millett KC, Sulkowski P, Sulkowska JI (2015) KnotProt: a database of proteins with knots and slipknots. Nucleic Acids Res. 43:D306–D314.

4. wwPDB consortium (2019) Protein Data Bank: the single global archive for 3D macromolecular structure data. Nucleic Acids Res 47:D520–D528.

5. Krissinel E, Henrick K (2004) Secondary-structure matching (SSM), a new tool for fast protein structure alignment in three dimensions. Acta Cryst D 60:2256–2268.

6. Krissinel E, Henrick K Multiple Alignment of Protein Structures in Three Dimensions. In: R. Berthold M, Glen RC, Diederichs K, Kohlbacher O, Fischer I, editors. Computational Life Sciences. Lecture Notes in Computer Science. Berlin, Heidelberg: Springer; 2005. pp. 67–78.

7. Perlinska AP, Niemyska WH, Gren BA, Rubach P, Sulkowska JI (2022) New 63 knot and other knots in human proteome from AlphaFold predictions. :2021.12.30.474018. Available from: https://www.biorxiv.org/content/10.1101/2021.12.30.474018v1

8. Colovos C, Yeates TO (1993) Verification of protein structures: patterns of nonbonded atomic interactions. Protein Sci 2:1511–1519.

9. Anon SAVESv6.0 - Structure Validation Server. Available from: https://saves.mbi.ucla.edu/

10. Jumper J, Evans R, Pritzel A, Green T, Figurnov M, Ronneberger O, Tunyasuvunakool K, Bates R, Žídek A, Potapenko A, et al. (2021) Highly accurate protein structure prediction with AlphaFold. Nature 596:583–589.

11. Varadi M, Anyango S, Deshpande M, Nair S, Natassia C, Yordanova G, Yuan D, Stroe O, Wood G, Laydon A, et al. (2022) AlphaFold Protein Structure Database: massively expanding the structural coverage of protein-sequence space with high-accuracy models. Nucleic Acids Research 50:D439–D444.

12. Anon AlphaFold Protein Structure Database. Available from: https://alphafold.ebi.ac.uk/
